# The role of vascular and lymphatic networks in bone and joint homeostasis and pathology

**DOI:** 10.3389/fendo.2024.1465816

**Published:** 2024-09-11

**Authors:** Jingxiong Huang, Chengcheng Liao, Jian Yang, Liang Zhang

**Affiliations:** ^1^ Center of Stomatology, West China Xiamen Hospital of Sichuan University, Xiamen, Fujian, China; ^2^ State Key Laboratory of Oral Diseases & National Center for Stomatology & National Clinical Research Center for Oral Diseases, West China Hospital of Stomatology, Sichuan University, Chengdu, Sichuan, China; ^3^ Department of Orthodontics II, Affiliated Stomatological Hospital of Zunyi Medical University, Guizhou, Zunyi, China; ^4^ Department of Oral Implantology, West China Hospital of Stomatology, Sichuan University, Chengdu, Sichuan, China

**Keywords:** vascular system, lymphatic system, bone, joint, homeostasis, disease

## Abstract

The vascular and lymphatic systems are integral to maintaining skeletal homeostasis and responding to pathological conditions in bone and joint tissues. This review explores the interplay between blood vessels and lymphatic vessels in bones and joints, focusing on their roles in homeostasis, regeneration, and disease progression. Type H blood vessels, characterized by high expression of CD31 and endomucin, are crucial for coupling angiogenesis with osteogenesis, thus supporting bone homeostasis and repair. These vessels facilitate nutrient delivery and waste removal, and their dysfunction can lead to conditions such as ischemia and arthritis. Recent discoveries have highlighted the presence and significance of lymphatic vessels within bone tissue, challenging the traditional view that bones are devoid of lymphatics. Lymphatic vessels contribute to interstitial fluid regulation, immune cell trafficking, and tissue repair through lymphangiocrine signaling. The pathological alterations in these networks are closely linked to inflammatory joint diseases, emphasizing the need for further research into their co-regulatory mechanisms. This comprehensive review summarizes the current understanding of the structural and functional aspects of vascular and lymphatic networks in bone and joint tissues, their roles in homeostasis, and the implications of their dysfunction in disease. By elucidating the dynamic interactions between these systems, we aim to enhance the understanding of their contributions to skeletal health and disease, potentially informing the development of targeted therapeutic strategies.

## 1 Introduction

Mammals possess two crucial vascular systems that support essential life functions. The first is the blood vessel system, responsible for delivering oxygen and nutrients to tissues and cells while removing metabolic waste. The second is the lymphatic vessel system, which manages the drainage of interstitial fluid and immune regulation. Though these systems develop independently, they are functionally and structurally interrelated. Their coordinated interaction is vital for maintaining the microcirculatory environment’s homeostasis. Recent studies have highlighted that blood and lymphatic vessels in bones and joints play multiple roles in maintaining the skeletal system’s homeostasis under both physiological and pathological conditions (1–5).

A specific subtype of blood vessels, known as type H blood vessels, is crucial for bone homeostasis. Characterized by high expression of CD31 and endomucin, these vessels support osteoprogenitors by coupling angiogenesis and osteogenesis (6, 7). Additionally, endothelial cells in type H blood vessels facilitate bone tissue homeostasis and regeneration through paracrine signaling (8, 9). Conversely, interruptions in blood flow and ischemia in the subchondral bone can impede nutrient diffusion to articular cartilage, resulting in bone cell death, joint damage, and conditions like arthritis (5, 10). Recent advancements have elucidated the functional role of the lymphatic network in the skeletal system. Studies have identified a lymphatic network in bones and the role of lymphangiocrine signaling in repairing radiation-induced bone injuries (2, 11, 12). Increasing evidence also points to the critical role of lymphatic vessels in maintaining joint homeostasis, with their pathological changes closely linked to the onset and progression of inflammatory joint diseases (4, 13). Consequently, the interest in the role of lymphatic vessels in tissue injury has grown. As research continues to evolve, the significance of the vascular-lymphatic network in bone tissue repair and joint diseases is increasingly recognized. This network maintains tissue homeostasis and controls disease progression by regulating local inflammatory activity, facilitating material exchange, and releasing paracrine signals from vascular and lymphatic secretions. Despite their importance, review on the holistic role of these networks in bone and joint homeostasis and related diseases remains limited.

In this review, we provide a comprehensive overview of the current knowledge on the structural and functional aspects of blood and lymphatic vessels in bone and joint. We summarize the regulatory effects of angiogenesis and endothelial secretory signals, as well as lymphogenesis and lymphatic secretory signals, on the homeostasis of bone and joint tissues under specific conditions. Additionally, we review the interactions and co-regulatory mechanisms of the vascular and lymphatic networks in these tissues. Finally, we examine the changes and potential regulatory mechanisms of these networks under pathological conditions.

## 2 Blood and lymphatic vessels in bone and joint

### 2.1 Blood vessels in bone and joint

The skeleton contains a complex vascular network essential for tissue oxygenation and metabolism. Blood vessels in bone play multiple roles in maintaining bone homeostasis under both physiological and pathological conditions. Studies have revealed diverse vascular subtypes and a specialized vascular microenvironment within bone.

**Figure 1:**
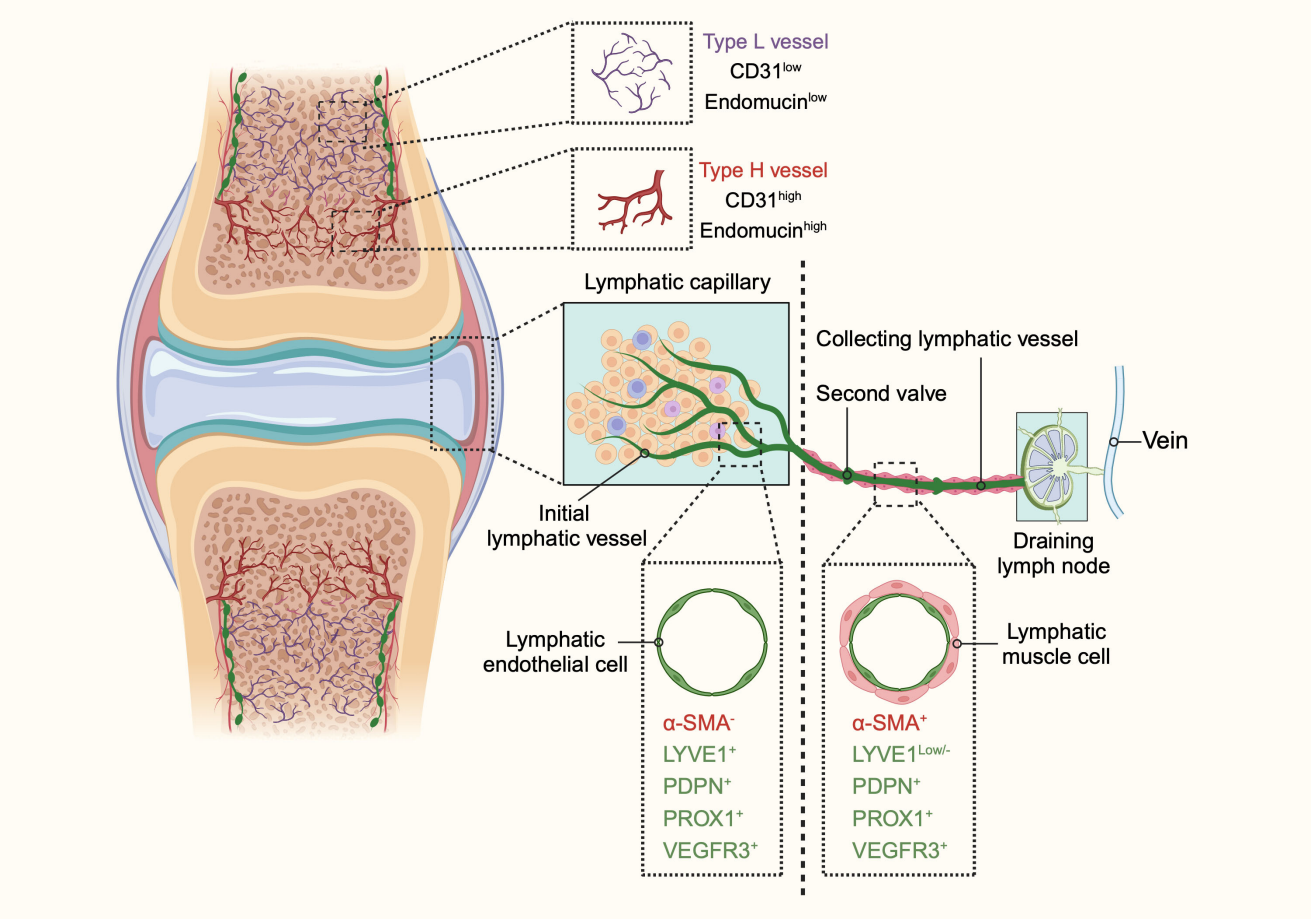
The schematic diagram illustrating the structure and distribution of blood vessels and lymphatic vessels in bones and joints. In bones, two distinct types of blood vessels are identified: type H and type L. Type H vessels, characterized by high expression of endomucin (Emcn) and cluster of differentiation 31 (CD31), are organized in a columnar manner with arterial connections and are primarily found in the metaphysis. Conversely, type L vessels, with lower levels of Emcn and CD31, are sinusoidal and located in the diaphysis. The identification of these vascular subtypes enhances our understanding of the heterogeneity of bone vasculature and its potential role in bone function in both health and disease. The lymphatic system in bones and joints also displays a hierarchical structure. Lymphatic vessels are present in the cortical regions and bone marrow cavity, with a higher concentration in the cortical areas. In joints, the lymphatic system begins with lymphatic capillaries, also known as initial lymphatic vessels. These vessels collect lymph and direct it towards collecting lymphatics equipped with anti-flowback valves. The lymph is then transported to draining lymph nodes before entering the venous system. Initial lymphatic vessels consist of a single layer of lymphatic endothelial cells (LECs) with a discontinuous basal lamina. In contrast, collecting lymphatic vessels are composed of tightly connected LECs, forming zipper-like junctions, and are surrounded by lymphatic muscle cells (LMCs) that facilitate lymph movement through contractions. Initial lymphatic vessels are marked by positive expression of lymphatic vessel endothelial hyaluronan receptor 1 (LYVE1), podoplanin (PDPN), prospero homeobox 1 (PROX1), and vascular endothelial growth factor receptor 3 (VEGFR3), but do not contain α-smooth muscle actin (α-SMA)-positive muscle cells. Collecting lymphatic vessels, however, exhibit lower levels of LYVE1 and positive expression of α-SMA, PDPN, PROX1, and VEGFR3. This differentiation between initial and collecting lymphatic vessels highlights their distinct roles and structures within the lymphatic system of bones and joints. (This figure is supported by Biorender).

Bone tissue features a unique type of blood vessel known as type H vessels. Predominant in the metaphysis and endosteum, these capillaries are characterized by high expression of CD31 (platelet and endothelial cell adhesion molecule 1) and endomucin (Emcn) (6, 7). These columnar vessels are interconnected at their distal ends near the growth plate in the metaphysis by structures termed loops or arches (7, 14, 15). Within the bone marrow cavity, there is a highly branched and relatively irregular sinusoidal vasculature with low expression of CD31 and Emcn, referred to as type L vessels (7, 16, 17). The base of the type H capillary columns in the metaphysis connects to the bone marrow vasculature at the metaphyseal–diaphyseal interface, linking the metaphysis to the diaphysis (15, 18). Sinusoidal and columnar vessels are interconnected, forming a single vascular network (**Figure 1**).

Type H vessels are less abundant than type L vessels due to their limited distribution and the large area of the bone marrow cavity (7). Fed directly by arterioles, type H vessels exhibit higher partial pressure of oxygen and blood flow than type L vessels (15, 19, 20). The lower permeability of type H vessels and nearby arterioles creates an environment low in reactive oxygen species (ROS) (16, 21). Conversely, the lower blood flow in sinusoidal type L vessels promotes the transendothelial migration of blood cells and the trafficking of leukocytes (21–23). Type L vessels in the bone marrow play crucial roles in hematopoietic cell trafficking and serve as vascular niches for myelopoiesis.

The differing properties of type H and type L vessels have significant functional consequences for tissue microenvironments. Type H and type L vessels also have distinct gene expression profiles, supporting different perivascular cell types and further impacting the local microenvironment (7). At the protein and transcriptome levels, in addition to CD31 and Emcn, type H vessels express various growth factors, including fibroblast growth factor 1 (FGF1), platelet-derived growth factor A (PDGF-A), and PDGF-B (7, 24). Endothelial cell transcripts that highly express CD31 and Emcn also express bone morphogenetic protein (BMP) family members BMP1, BMP4, and BMP6, known to promote bone formation (24). This may explain the presence of osteoprogenitor cells around type H capillaries. Type H vessels are closely associated with osteoprogenitor cells, contributing significantly to bone remodeling and regeneration. The Notch ligand DLL4, an important regulator of angiogenesis, is also highly expressed in type H vessels adjacent to the growth plate (6). Given their functional nature and gene expression profile, type H endothelial cells likely contribute to the coupling of angiogenesis and osteogenesis in bone development.

### 2.2 Lymphatic vessels in bone and joint

The lymphatic system plays a crucial role in regulating interstitial fluid homeostasis, waste clearance, and immune cell surveillance (25, 26). Through a network of initial lymphatic capillaries and collecting lymphatic vessels, the system transports interstitial fluid from peripheral tissues to lymph nodes, enters the right lymphatic duct and thoracic duct, draining into the subclavian veins and returning to the blood circulation (27–29).

Initial lymphatic capillaries are lined by a single layer of lymphatic endothelial cells connected by specialized button-like junctions, which are highly permeable to solutes and macromolecules (30–32). When external compressive forces exceed the interluminal fluid pressure, interstitial fluid is pushed into the initial lymphatic capillaries and becomes lymph. Lymphatic endothelial cells overlap to form primary valves that prevent lymph backflow. These initial lymphatics converge to form larger collecting lymphatic vessels, which are connected by adjacent lymphatic endothelial cells through tight zipper-like junctions, making them less permeable than initial lymphatics (29, 31–34). Collecting lymphatic vessels are surrounded by one or more layers of lymphatic muscle cells (LMCs), which facilitate vessel contraction to propel lymph forward (35). These vessels also contain secondary bicuspid valves to prevent backflow (36). After collection by the lymphatic vessels, lymph traverses the afferent lymphatics to reach the draining lymph nodes (DLNs). The lymphatic sinuses within DLNs are highly organized structures containing immune cells, where adaptive immune responses are generated (37). Finally, lymph exits the nodes via efferent lymphatic vessels and reenters the circulatory system (**Figure 1**).

Lymphatic vessels are reported not to be present in several tissues, including the brain and eye (38, 39). The presence of lymphatics in bone has been a topic of considerable controversy. Traditionally, it was believed that bones and bone marrow lack lymphatic vessels, and that the growth of lymphatic vessels in bone could be detrimental, as observed in Gorham-Stout disease, a rare bone disorder characterized by the abnormal growth of lymphatic vessels in bones (40–42). Lymphatics in bone are not typically visible on routine lymphangiograms in humans, possibly due to the rare connections between deep and superficial lymphatics. Some historical studies, however, suggest a different conclusion. Injection of radio-opaque agents or macromolecular markers, such as ferritin and horseradish peroxidase, into the bone marrow has been shown to reach the periosteal surface of the bone (43–46). These findings imply the potential presence of lymphatic channels in bone. These results indicate that if macromolecules flow from the bone marrow to the periosteal surface, as some fluid transport studies suggest, this may occur through an alymphatic system or involve matrix prelymphatics and perivascular prelymphatics lacking an endothelial lining, similar to those described in the eye and brain (45, 47). It could be argued that, due to the relatively large gaps between the pseudopodial processes of endothelial cells in bone sinusoids, the free movement of macromolecules and newly formed blood cells between the extravascular and intravascular compartments is possible, negating the need for a lymphatic system for fluid transport in bone. However, recent research has revealed the presence of lymphatic vessels in long bones, the dura mater of the mouse brain, and the spinal vertebral column (12, 48, 49). Notably, Biswas et al., using high-resolution light-sheet imaging and cell-specific mouse genetics, demonstrated the presence of lymphatic vessels in mouse and human bones and further validated the importance of lymphatic endothelial cell-derived secretory proteins for bone regeneration (2). In addition, it has been found that lymphatic vessels were identified within the stratified connective tissues surrounding the fetal cartilaginous knee joint tissues in the fetus and adult mice, but not detected in cartilage tissues (50–52). Moreover, lymphatic vessels have been identified within the periosteum of long bones (52). Therefore, lymphatic vessels are extensively distributed throughout the various tissues of the bone and joint, excluding articular cartilage (**Figure 1**).

## 3 The dynamic interplay of vascular and lymphatic endothelial cells in development

The vascular-lymphatic network is essential for maintaining fluid homeostasis, supporting tissue repair, and facilitating immune cell trafficking. Understanding the biology of endothelial cells (ECs), which form the lining of blood and lymphatic vessels, is fundamental to these interactions. EC populations are regulated by a complex network of signaling pathways that govern their spatial and temporal organization during critical events in development, growth, and regeneration.

Both blood endothelial cells (BECs) and lymphatic endothelial cells (LECs) originate from primitive vascular endothelial progenitor cells derived from the mesoderm (53, 54). This common origin gives rise to BECs through the process of vasculogenesis, driven by key factors such as ETS variant transcription factor 2 (ETV2), fibroblast growth factor 2 (FGF2), bone morphogenetic protein 4 (BMP4), and Indian hedgehog signaling molecule (IHH) (55–59). Angioblasts formed through these pathways further mature via vasculogenesis or angiogenesis, with vascular endothelial growth factor A (VEGFA) playing a significant role in promoting the sprouting of new vessels (60).

The differentiation of these progenitor cells into either BECs or LECs is governed by distinct yet overlapping signaling mechanisms. In the early stages of development, ECs undergo arterial-venous specification, where arterial and venous fates are distinguished by the expression of EFNB2 and EPH receptor B4 (EPHB4), respectively (61). The VEGFA-VEGFR2 signaling pathway is crucial for promoting arterial phenotypes while inhibiting venous characteristics (62, 63). The Notch signaling pathway, activated by VEGF, enhances arterial gene expression and suppresses venous patterning, with Wnt signaling regulating arterial specification upstream of Notch through β-catenin and Delta-like 4 (DLL4) expression (64–69). Conversely, the acquisition of the venous phenotype involves nuclear receptor subfamily 2 (NR2F2, also known as COUP transcription factor 2, COUP-TFII), which suppresses Notch signaling and, along with VEGFA-VEGFR2 interactions, supports venous specification (65, 70–72). The mitogen-activated protein kinase (MAPK) pathway promotes arterial specification under VEGFR2 activation, while VEGFR2 also activates phosphoinositide-3-kinase (PI3K)/AKT to facilitate venous specification (73).

LECs primarily originate from venous ECs through transdifferentiation, though non-venous ECs also contribute (27). During embryonic development, venous ECs in the dorsolateral region of the cardinal vein sprout to initiate lymphangiogenesis, regulated by a network of signals including NR2F2. Deficiency in NR2F2 disrupts lymphangiogenesis and leads to edema, indicating its critical role in lymphatic specification (74, 75). Prospero homeobox protein 1 (PROX1), a classical marker for LECs, is essential for initiating lymphatic specification. In venous ECs, NR2F2 maintains the venous phenotype by suppressing Notch signaling, while in LECs, the NR2F2-PROX1 heterodimer reverses this suppression (76). SRY-related HMG-box 18 (SOX18) also enhances PROX1 transcription, driving LEC specification through a positive feedback loop (77). VEGFC, working alongside transcription factors such as SOX7 and MAFB (musculoaponeurotic fibrosarcoma oncogene homolog B), further promotes lymphatic specification by upregulating multiple LEC markers, including PROX1 (78, 79).

During the life cycle, ECs exhibit phenotypic plasticity, undergoing transdifferentiation under specific conditions. This adaptability is exemplified in the development of capillaries and the transdifferentiation of venous ECs into arterial ECs and LECs. Such transitions are regulated by signaling pathways like VEGF, Notch, and Wnt, which orchestrate the formation and specialization of blood and lymphatic networks. Understanding the dynamic interplay between these networks is crucial for developing targeted therapies. While the blood and lymphatic systems are relatively independent, they regulate and promote each other’s development. Disruption in this mutual regulation can lead to developmental abnormalities, underscoring the importance of their interdependent signaling mechanisms during growth and regeneration.

## 4 Vascular-lymphatic network in skeletal healing

### 4.1 Angiogenesis and angiocrine modulation in skeletal healing

The significant changes in BECs during tissue repair closely resemble those observed in development. Angiogenesis is the primary mechanism for new blood vessel formation in response to injury. Unlike the homeostatic state, repair in the context of injury has distinct characteristics. This shift is linked to increased local inflammation following injury, which activates angiogenesis to aid in vascular network regeneration (80). In bone, a unique form of angiogenesis, termed vessel bulging angiogenesis, is prominent, with type H vessels playing a crucial role. These vessels facilitate revascularization in hard tissue injuries, including spinal fusion surgeries (81), tooth extraction wounds (82), and diabetic osteoporosis (83). Type H vessels form by merging vascular buds from opposite ends, and although typical tip cells are not observed in bone angiogenesis, ECs display tip cell-like features such as filopodia and directional migration along VEGF gradients (6, 84, 85). Notably, Notch signaling is strongly activated during bone vascular regeneration, contrasting with its inhibitory role in vessel sprouting in other tissues. The intensity of Notch signaling correlates with blood flow rate and restoring blood flow in aging individuals promotes bone healing (7, 15). The new vascular network invades the injury site, restores blood supply, and provides channels for osteoblast precursors, coupling angiogenesis with osteogenesis (86).

BECs also release angiocrine factors, influencing vascular modulation during injury (87). In the skeletal system, type H vessels release factors like Noggin, which regulate skeletal morphology and ossification (88). Additionally, type H ECs release matrix metallopeptidases (MMPs) to remodel the extracellular matrix (ECM), essential for cartilage resorption during bone remodeling (89, 90). Various pro-angiogenic and angiocrine factors, including VEGFA, FGF2, and FGF9, are involved in inducing vascularization and bone growth during repair. VEGFA promotes bone repair, while VEGFR1 negatively regulates blood vessel growth and fracture repair (91, 92). Placental growth factor (PIGF), a VEGFR1 ligand, facilitates bone healing (81, 93). FGF signaling, particularly FGF2 and FGF9, stimulates angiogenesis and osteogenesis during bone repair (6, 83, 84). Transforming growth factor beta (TGFβ), BMP-2, BMP-7, and growth differentiation factor (GDF) also enhance angiogenesis and osteogenesis during healing (86, 87). Angiocrine crosstalk via Notch signaling promotes fracture repair, as evidenced by reduced hematopoietic stem cell (HSC) regeneration following endothelial-specific deletion of the Notch ligand Jag1 (88, 90). ECs also upregulate factors like FGF2, BMP4, Insulin-like growth factor-binding protein 2 (IGFBP2), and Angiopoietin1, expanding hematopoietic stem progenitor cells (HSPCs) and contributing to hematopoietic recovery and bone repair after acute bone marrow injury, such as chemotherapy and irradiation (89, 94).

### 4.2 Lymphangiogenesis and lymphangiocrine modulation in skeletal healing

The lymphatic system is primarily responsible for transporting body fluids and immune cells. Beyond these canonical functions, lymphatic vessels are implicated in diverse physiological roles across various organs and tissues. Recent research highlights the correlation between the integrity of lymphatic vessels and several metabolic phenotypes, including insulin resistance, cardiovascular diseases, lipid absorption, and liver injuries (95–98). Notably, LECs are now recognized for their role in regulating metabolic homeostasis through the secretion of various proteins, referred to as lymphangiocrine signals.

Lymphangiogenesis, or the formation of new lymphatic vessels from existing ones, is especially important for bone and joint health. Research shows that lymphangiocrine signals significantly impact the aging process. Biswas et al. discovered lymphatic vessels within bones, confirming their role in bone regeneration (2). Advanced imaging revealed these vessels at a single-cell level, expanding in response to stress in a manner dependent on the inflammatory cytokine Interleukin 6 (IL-6). LECs were found to secrete C-X-C motif chemokine 12 (CXCL12), a chemokine that regulates blood cell production and bone healing. Remarkably, injecting LECs from young mice into aged mice restored both bone and blood cell regeneration, highlighting the crucial role of lymphangiocrine signals in aging (2). The results of study highlight the importance of lymphangiocrine signals for metabolic homeostasis.

The lymphatic system also plays a vital role in managing inflammation, particularly in conditions like rheumatoid arthritis (RA), which is marked by chronic joint inflammation and progressive damage. In RA, inflammatory cytokines such as TNF-α, IL-1, and IL-6 trigger synovial inflammation, leading to joint pain, swelling, and functional impairment (99–101). Lymphatic vessels are crucial for clearing these inflammatory cells and mediators from the inflamed synovium. Studies using animal models of RA have shown that the VEGF-C/VEGFR3 signaling pathway is vital for lymphangiogenesis in arthritis. VEGF-C and its receptors, VEGFR3 and VEGFR2, are highly expressed in arthritic synovial tissue, promoting the growth and migration of LECs (102–104). Macrophages in the inflamed environment also express VEGF-C and VEGFR3, further supporting lymphangiogenesis (105).

### 4.3 Co-regulation of blood and lymphatic endothelial cells: VEGF and BMP signaling pathways

**Figure 2:**
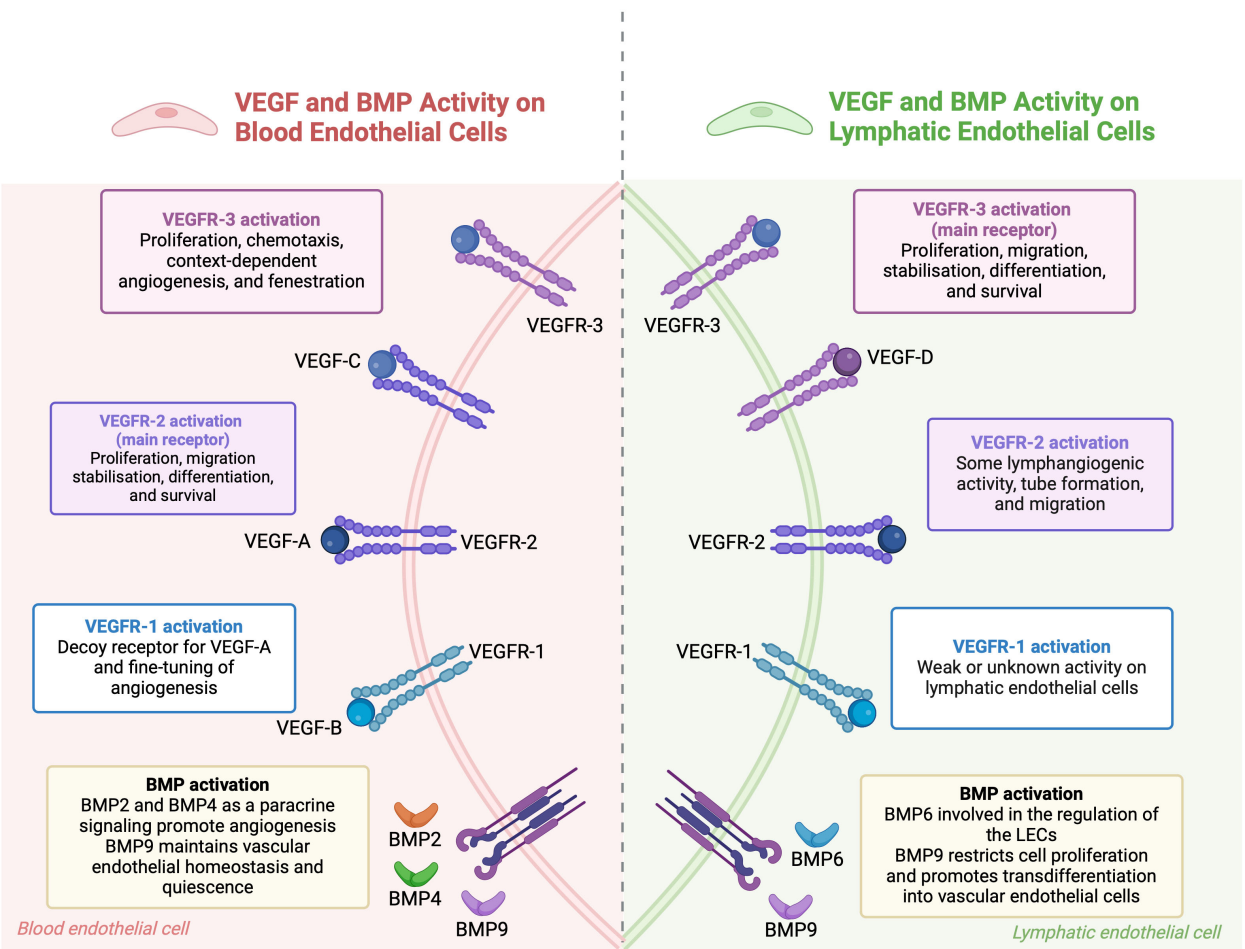
The combined roles of VEGF and BMP pathways in regulating endothelial cells. In blood endothelial cells, VEGF-A binds to VEGFR-1 and VEGFR-2 receptors. VEGFR-1 modulates angiogenesis, while VEGFR-2 promotes cell growth, movement, stabilization, differentiation, and survival. BMP signaling, through BMP2, BMP4, and BMP9, supports blood vessel formation and maintains vascular stability. In lymphatic endothelial cells, VEGF-C and VEGF-D primarily activate VEGFR-3, leading to cell growth and movement, while VEGFR-2 also aids in lymphatic vessel formation. BMP6 and BMP9 regulate lymphatic endothelial cells, with BMP9 encouraging their transformation into blood vessel cells. BMP signaling pathways interact with VEGF pathways to maintain endothelial cell function and regulate angiogenesis. (This figure is supported by Biorender).

The co-regulation of BECs and LECs by shared signaling pathways underscores their interdependence. This knowledge has clinical potential, especially in precision medicine, where manipulating these pathways could lead to novel treatments for vascular and lymphatic disorders. VEGF and BMP are key regulators of both blood and lymphatic vessels, orchestrating their development, function, and homeostasis (106, 107) (**Figure 2**).

The VEGF family is central to the repair and regeneration of the vascular-lymphatic network post-injury. VEGFA-VEGFR2 and VEGFC-VEGFR3 are primary signals for BECs and LECs, respectively, driving their migration, proliferation, and participation in regeneration. However, VEGFR2 can also be expressed in LECs, and VEGFC can bind to VEGFR2 in BECs, indicating the complexity of VEGF signaling in this network. VEGFC not only induces lymphangiogenesis but also stabilizes blood and lymphatic capillaries by regulating PDGF-B expression, which recruits pericytes and lymphatic smooth muscle cells to the vessels (108, 109). Complementary mechanisms refine vascular-lymphatic network regulation. For example, receptor activity-modifying protein 1 (RAMP1) has been shown to promote both angiogenesis and lymphangiogenesis in skin wounds, with its absence leading to impaired wound healing due to reduced VEGFA and VEGFC levels (110). Additionally, Ras homolog family member B (RHOB) and vascular endothelial zinc finger 1 (VEZF1) have opposing roles in vessel growth. RHOB inhibits blood vessel growth while promoting lymphatic vessel growth (111). VEGFR1, previously considered a decoy receptor, has been found to promote angiogenesis and lymphangiogenesis by modulating the secretome of pro-inflammatory macrophages in diabetes-related delayed wound healing models (60, 112, 113). Hemostasis also couples blood-lymphatic vessels post-injury by releasing coagulation proteases that cleave VEGFC and VEGFD, promoting LEC proliferation. Activated platelets further facilitate VEGFC-VEGFR3 binding by upregulating VEGFR3 expression in LECs (114). Angiopoietins also play a role in co-regulation, promoting angiogenesis and lymphangiogenesis at wound margins and influencing vascular-lymphatic remodeling during inflammation, though their effects can vary depending on the context (115, 116).

BMPs are crucial regulators of both blood and lymphatic vessels. In the blood vasculature, BMP2, BMP4, BMP9, and BMP10 play crucial roles. BMP2 and BMP4 are produced locally and act as paracrine signals, promoting angiogenesis. In contrast, BMP9 and BMP10 circulate systemically and inhibit sprouting (117, 118). BMP9 and BMP10 are particularly important for vessel stabilization and quiescence, inhibiting excessive sprouting and maintaining endothelial homeostasis (119, 120). In the lymphatic system, BMP6 and BMP9, circulating in the systemic bloodstream, signal to LECs. BMP9 is especially crucial for the maturation of lymphatic vessels and the formation of lymphatic valves. BMP9 knockout mice exhibit defects such as dilated lymphatic vessels and a reduced number of valves. BMP9, through the ALK1 receptor, regulates key genes like Lyve1, Foxc2, Connexin37, Ephrin-b2, and Neuropilin1, which are essential for lymphatic valve development (121, 122). Moreover, BMP9 downregulates PROX1 in LECs, leading to restricted cell proliferation and a trans-differentiation of lymphatic endothelial cells to blood endothelial cells (123, 124). BMP2 also plays a role in the lymphatic system by negatively regulating lymphatic vessel development. It inhibits PROX1 expression and induces miR-31 and miR-181a, which target Prox1 and impede lymphatic endothelium specification (125).

## 5 Maladaptation of vascular-lymphatic network in skeletal disease

### 5.1 Inflammation

Under inflammatory stress, the vasculature in the bone marrow is crucial for supporting bone remodeling. ECs express BMP-2, promoting bone formation, and release osteoprotegerin (OPG) to reduce osteoclastogenesis during diabetes (126, 127). Additionally, multiple cytokines such as IL-6, TNF-α, and IFN-γ are produced by ECs under inflammatory conditions. These cytokines activate Nuclear factor kappa-light-chain-enhancer of activated B cells (NF-κB) signaling, which regulates hematopoietic stem and progenitor cell (HSPC) functions. Inhibiting the endothelial NF-κB pathway improves HSPC proliferation and hematopoietic recovery following myelosuppressive injury (128). IL-33, a pro-inflammatory cytokine produced by CD105-expressing ECs, promotes the differentiation of bone marrow-derived stromal cells into osteoblasts and enhances calcium deposition (129).

Recent studies highlight the connection between lymphangiocrine signaling and inflammation. Under inflammatory conditions, lymphatic vessels within the bone undergo significant changes, including increased lymphangiogenesis and elevated expression of specific cytokines and growth factors that support this expansion and associated immune responses (2). IL-6 drives lymphangiogenesis in bones, and the secretion of CXCL12 from proliferating LECs is critical for hematopoietic and bone regeneration. Moreover, lymphangiocrine CXCL12 triggers the expansion of mature Myh11+ CXCR4+ pericytes, which differentiate into bone cells and contribute to bone and hematopoietic regeneration. In aged animals, this expansion of lymphatic vessels and Myh11-positive cells in response to genotoxic stress is impaired (2). The increased presence of lymphatic vessels and activated LECs significantly impacts bone regeneration and repair processes.

### 5.2 Osteoarthritis

OA is a common joint disorder characterized by the degeneration of articular cartilage and inflammation of surrounding tissues due to aging-related mechanical degradation and subchondral bone disorders (130, 131). Synovial cells play a crucial role in OA by releasing inflammatory mediators that stimulate the production of inflammatory cytokines and matrix-degrading enzymes like MMPs and ADAMTS (a disintegrin and metalloproteinase with thrombospondin motifs) proteins in chondrocytes, leading to cartilage destruction (132, 133). Immunohistochemical analysis of synovial specimens from OA patients reveals increased lymphatic vessels infiltrated by inflammatory cells, indicating their involvement in OA pathogenesis (134). Transmission electron microscopy has shown dysfunction in microcirculation and lymphatic drainage in OA patients (135). Post-mortem analysis of knee synovium sections from OA patients shows reduced lymphatic vessel density, negatively correlated with synovial effusion, suggesting impaired lymphatic drainage exacerbates joint inflammation (136). Dynamic changes in lymphatic structure and function may significantly impact OA progression, warranting further investigation. In a mice model of meniscal-ligamentous injury (MLI)-induced OA, increased capillary lymphatics and decreased collecting lymphatic vessels were observed (50). Although lymphatic capillaries increased, their drainage function declined due to a leaky phenotype. This led to impaired lymphatic pumping and accumulation of pro-inflammatory factors in OA-affected knees, supported by findings in human OA samples (52, 137). These results indicate impaired synovial lymphatic drainage during OA progression.

Macrophages also play a significant role in joint inflammation and bone destruction in OA, potentially through interaction with lymphatic vessels (138, 139). Macrophages can be categorized into pro-inflammatory M1 and anti-inflammatory M2 phenotypes, accumulating and polarizing within the synovium and articular cavity during OA progression. Early-stage OA shows synovitis and M1 macrophage accumulation near lymphatic vessels, with M1 macrophages promoting destructive processes by regulating inflammatory mediators like TNF, IL-1, and iNOS (140). Itch, a negative regulator of the NF-κB pathway, suppresses pro-inflammatory macrophage polarization and IL-1α release (141, 142). Knockout of itch in mice results in severe OA phenotypes and impaired lymphatic drainage due to M1 macrophage-induced inflammation (143). Decreased FGFR3 expression in OA patient monocytes, and conditional FGFR3 knockout in macrophages exacerbates joint destruction through synovitis and macrophage accumulation via CXCL12/CXCR7-dependent chemotaxis (144). Since CXCL12 from LECs is crucial for tissue regeneration post-injury, further research is needed on the interplay between synovial macrophages and lymphatic vessels in OA.

### 5.3 Rheumatoid arthritis

RA is a chronic autoimmune disorder primarily affecting the joints, leading to persistent inflammation and progressive damage. This inflammation triggers the release of inflammatory mediators and activates immune cells, further worsening the condition. Inflamed joints in RA patients exhibit a significant increase in activated and infiltrated immune cells, such as macrophages, lymphocytes, and plasma cells. These cells are crucial in the progression of joint inflammation as they produce and release various mediators, including cytokines, chemokines, and enzymes (100, 101). Key cytokines involved in RA pathogenesis include tumor necrosis factor TNF-α, IL-1, and IL-6. These cytokines induce synovial inflammation and vasodilation, resulting in joint pain, swelling, and functional impairment (99). RA is also characterized by increased angiogenesis and vessel density in non-calcified articular cartilage regions. Lymphocyte infiltration and active ECs are essential for the trafficking of leukocytes into the joint during RA progression (145). Intercellular adhesion molecule-1 (ICAM-1), vascular cell adhesion protein 1 (VCAM-1), and E-selectin expressed by ECs promote the migration of leukocytes and fibroblasts into RA joints (146). Specifically, endothelial Notch3 signaling drives the differentiation of synovial fibroblasts, which acquire an invasive phenotype during the disease (147). This invasive behavior of synovial fibroblasts contributes to the overall joint damage and functional decline observed in RA patients.

Clinical studies and animal models indicate that lymphatic vessels likely play a crucial role in clearing inflammatory cells and mediators from the inflamed synovium. The VEGF family comprises key regulators in angiogenesis and lymphangiogenesis, including VEGF-A, VEGF-B, VEGF-C, VEGF-D, VEGF-E, VEGF-F, and placenta growth factor (PIGF) (104). These VEGF ligands activate signaling pathways by binding to tyrosine kinase receptors known as vascular endothelial growth factor receptors (VEGFRs), which have three subtypes: VEGFR1, VEGFR2, and VEGFR3. While VEGFR1 and VEGFR2 primarily regulate angiogenesis, VEGFR3 signaling is central to lymphangiogenesis (102). The downstream signaling pathways activated by VEGF-C/VEGFR3 include mitogen-activated protein kinase/extracellular signal-related kinase (MAPK/ERK), phosphatidylinositol 3-kinase/protein kinase B (PI3k/AKT), and Jun N-terminal kinase1/2 (JNK1/2) pathways (148, 149). Activation of these pathways leads to the proliferation, survival, and migration of LECs and the remodeling of lymphatic vessels. Previous studies have shown high expression of VEGF-C and its receptors, VEGFR2 and VEGFR3, in the synovial tissues of arthritis patients compared to healthy controls. Macrophages also exhibit high expression of VEGF-C and VEGFR3. Additionally, significantly elevated levels of VEGF-C have been observed in the synovial fluid of patients with RA, showing a strong positive correlation with TNF-α levels (150).

### 5.4 Bone metastasis

Bone metastasis is a frequent complication of several primary tumors, where disseminated tumor cells (DTCs) can remain dormant for extended periods before reactivation and metastatic growth (151, 152). The process of reactivation and metastasis is intricately linked to the vascular and lymphatic networks within the bone microenvironment.

ECs in the bone marrow play crucial roles in both supporting and regulating DTC behavior. They produce thrombospondin-1, inducing DTC quiescence, and express molecules like Von Willebrand factor (VWF) and vascular cell adhesion molecule 1 (VCAM-1), which affect DTC interaction with the perivascular niche and chemotherapy sensitivity (153, 154). Moreover, ADAM17-regulated CX3CL1 expression by bone marrow ECs promotes specific types of metastases, such as spinal metastasis from hepatocellular carcinoma (155). The vascular structure within bones also influences metastatic progression. Sinusoids and low blood flow facilitate interactions between tumor cells and ECs, while type H vessels with higher blood flow and oxygen supply support tumor cell survival and resistance to therapies (154). Reduction in blood flow diminishes type H vessels and inhibits pericyte expansion, thereby rendering DTCs more susceptible to treatment (156).

Tumors undergo phenotypic changes through accumulated genetic mutations, fostering polyclonal cell populations. Epithelial-mesenchymal transition (EMT) enhances cancer cell motility and invasiveness, mediated by cytokines like TGF-β, FGF, and others. EMT also reduces E-cadherin expression and promotes mesenchymal markers like vimentin and N-cadherin, enhancing malignant traits and chemotherapy resistance (157, 158). In the context of lymphatic involvement, tumor cells invade lymphatic vessels primarily from the peritumoral regions rather than from within the tumor itself due to high interstitial pressure (159). TGF-β signaling and ALK5 inhibitors play significant roles in tumor lymphangiogenesis in tumor xenografts (160, 161). Studies suggest that TGF-β influences tumor metastasis by regulating the structure and function of newly formed tumor lymphatic vessels. Secondary lymphedema, a common complication of cancer treatment, often involves increased TGF-β1 levels. In mouse models, inhibition of TGF-β1 has been demonstrated to mitigate the severity of lymphedema (162). Thus, targeting TGF-β could potentially effectively inhibit lymphatic metastasis and reduce lymphedema.

Several studies emphasize the significant role of bone marrow mesenchymal stem cells (BM-MSCs) in cancer progression, particularly through their impact on lymphangiogenesis (163–165). Human BM-MSCs contribute to tumor growth and metastasis by promoting both neovascularization and the formation of lymphatic vessels (164). Research shows that BM-MSCs and their conditioned medium not only support tumor growth but also facilitate lymph vessel formation in metastatic environments by increasing the expression of lymph-associated markers and enhancing tube formation in lymphatic endothelial cells and specific tumor cell lines (165). However, there are concerns about the potential for these processes to awaken dormant tumors through lymphangiogenesis. Additionally, both human and murine BM-MSCs have demonstrated the ability to adopt a lymphatic phenotype and stimulate lymphatic vessel formation by secreting factors like VEGF-A (163). This factor activates the VEGFR-2 pathway in lymphatic endothelial cells (LECs), leading to increased LEC proliferation, migration, and tube formation, which, in turn, enhances lymphatic vessel density within tumors and promotes metastasis (163). While these findings suggest promising therapeutic applications of MSCs in regenerative medicine, they also highlight the need to consider their role in cancer-related lymphangiogenesis when developing cancer treatment strategies.

Overall, understanding the maladaptation of the vascular-lymphatic network in bone metastasis involves deciphering complex interactions between tumor cells, endothelial cells, and the lymphatic system. Therapeutic strategies targeting these interactions hold promise for improving outcomes in patients with metastatic bone disease, necessitating further research into the precise molecular mechanisms driving vascular and lymphatic dysregulation in this context.

## 6 Targeting the vascular-lymphatic network as a potential therapeutic strategy

The vascular and lymphatic networks play crucial roles in maintaining tissue homeostasis and responding to pathological conditions in bone and joint disorders. Given their involvement in inflammation, tissue regeneration, and disease progression, targeting these networks presents a promising therapeutic approach. By modulating angiogenesis and lymphangiogenesis, it is possible to address the underlying mechanisms of various bone and joint diseases, potentially leading to more effective treatments and improved patient outcomes.

Angiogenesis, the process of blood vessel formation, is essential for bone tissue engineering and regeneration. Strategies to enhance vascularization in engineered bone tissues have shown significant promise, particularly through the delivery of angiogenic growth factors such as VEGF, Angiogenin (ANG), and PDGF (166–169). For instance, the incorporation of VEGF into bone scaffolds has been demonstrated to promote neovascularization and bone healing, as evidenced by advanced bone regeneration in animal models (167). Additionally, the sustained release of these growth factors, facilitated by sophisticated delivery systems, ensures prolonged therapeutic effects, making them superior to bolus injections (166). Given the coupling of angiogenesis and osteogenesis, these strategies hold great potential for improving the success rates of bone tissue engineering and addressing bone-related pathologies.

The lymphatic network, particularly lymphangiogenesis and lymphatic drainage, also offers potential therapeutic targets, especially in conditions like RA and OA. The VEGF-C/VEGFR3 signaling pathway has emerged as a key regulator of lymphangiogenesis and lymphatic function. In RA, enhancing lymphatic drainage through intra-articular administration of VEGF-C has been shown to reduce joint damage by promoting local lymphatic function (170). Similarly, in OA, impaired lymphatic drainage has been linked to disease progression, and targeting VEGF-C/VEGFR3 signaling has demonstrated the potential to enhance lymphatic function and mitigate tissue damage (137, 171). Despite these promising findings, further research is necessary to fully understand the long-term effects and safety of such treatments, particularly in chronic conditions like arthritis.

VEGF serves as a crucial common regulator, linking both the vascular and lymphatic networks. VEGF not only drives angiogenesis, essential for blood vessel formation and bone regeneration, but also plays a significant role in lymphangiogenesis through its interaction with VEGF-C and the VEGFR3 signaling pathway (172–174). This dual role of VEGF highlights its importance as a therapeutic target that can simultaneously influence both blood and lymphatic vessel dynamics. By modulating VEGF activity, it may be possible to achieve coordinated regulation of these two networks, offering a unified approach to treating complex bone and joint disorders where both vascular and lymphatic dysfunctions are involved.

## 7 Conclusion and perspective

The intricate interplay between blood and lymphatic networks is vital for maintaining bone and joint homeostasis and responding to pathological conditions. Type H blood vessels play a crucial role in coupling angiogenesis with osteogenesis, while emerging evidence highlights the significance of lymphatic vessels in bone support regeneration after injury. These networks work synergistically to regulate bone homeostasis and facilitate bone repair. Understanding these interactions provides a comprehensive view of skeletal biology and offers insights into the mechanisms underlying bone and joint diseases. Future research should focus on elucidating the specific molecular pathways and signaling mechanisms driving these interactions, which could pave the way for novel therapeutic strategies. Additionally, integrating recent advancements in vascular and lymphatic biology will enhance our ability to develop targeted treatments for bone and joint diseases, ultimately improving patient outcomes. This evolving field holds promise for significant breakthroughs in both basic science and clinical applications.

Research on lymphatic vessels in bone tissue lags behind the more extensive studies on blood vessels in bones and joints. While it’s known that lymphatic vessels are present in bones and play roles in fluid transport and immune surveillance, their drainage pathways within bones remain unexplored. Identifying these drainage routes is crucial for a deeper understanding of bone physiology, the specific functions of lymphatic vessels in bone, and potential drug interventions. Further research using advanced techniques such as single-cell sequencing and lineage tracing is necessary to identify the key cell subsets and molecular characteristics of lymphatic vessels, particularly in disease conditions. Understanding how lymphatic vessels change and function during different stages of diseases like RA, OA, and aging could help pinpoint the optimal timing for clinical interventions. We anticipate that future research will lead to better strategies for regulating lymphatic vessels in joints, ultimately improving the treatment and outcomes of inflammatory joint diseases.

## References

[1] Meng YM Jiang X Zhao X Meng Q Wu S Chen Y. Hexokinase 2-driven glycolysis in pericytes activates their contractility leading to tumor blood vessel abnormalities. Nat Commun. (2021) 12:6011. doi: 10.1038/s41467-021-26259-y 34650057 PMC8517026

[2] BiswasLChenJDe AngelisJSinghAOwen-WoodsCDingZ. Lymphatic vessels in bone support regeneration after injury. Cell. (2023) 186:382–397.e24. doi: 10.1016/j.cell.2022.12.031 36669473

[3] Peng Y Kenney HM de Mesy Bentley KL Xing L Ritchlin CT Schwarz EM. Distinct mast cell subpopulations within and around lymphatic vessels regulate lymph flow and progression of inflammatory-erosive arthritis in TNF-transgenic mice. Front Immunol. (2023) 14:1275871. doi: 10.3389/fimmu.2023.1275871 38155962 PMC10752982

[4] Zhou S Zhao G Chen R Li Y Huang J Kuang L. Lymphatic vessels: roles and potential therapeutic intervention in rheumatoid arthritis and osteoarthritis. Theranostics. (2024) 14:265–82. doi: 10.7150/thno.90940 PMC10750203 38164153

[5] Tuckermann J Adams RH. The endothelium-bone axis in development, homeostasis and bone and joint disease. Nat Rev Rheumatol. (2021) 17:608–20. doi: 10.1038/s41584-021-00682-3 PMC7612477 34480164

[6] Ramasamy SK Kusumbe AP Wang L Adams RH. Endothelial Notch activity promotes angiogenesis and osteogenesis in bone. Nature. (2014) 507:376–80. doi: 10.1038/nature13146 PMC4943529 24647000

[7] Kusumbe AP Ramasamy SK Adams RH. Coupling of angiogenesis and osteogenesis by a specific vessel subtype in bone. Nature. (2014) 507:323–8. doi: 10.1038/nature13145 PMC4943525 24646994

[8] Liu X Zhang P Gu Y Guo Q Liu Y. Type H vessels: functions in bone development and diseases. Front Cell Dev Biol. (2023) 11:1236545. doi: 10.3389/fcell.2023.1236545 38033859 PMC10687371

[9] Xu Z Kusumbe AP Cai H Wan Q Chen J. Type H blood vessels in coupling angiogenesis-osteogenesis and its application in bone tissue engineering. J BioMed Mater Res B Appl Biomater. (2023) 111:1434–46. doi: 10.1002/jbm.b.35243 36880538

[10] Wang M Wu Y Li G Lin Q Zhang W Liu H. Articular cartilage repair biomaterials: strategies and applications. Mater Today Bio. (2024) 24:100948. doi: 10.1016/j.mtbio.2024.100948 PMC10806349 38269053

[11] McCarter AL Khalid A Yi Y Monroy M Zhao H Rios JJ. Bone development and fracture healing is normal in mice that have a defect in the development of the lymphatic system. Lymphology. (2020) 53:162–71. PMC8489280 33721923

[12] Jacob L Boisserand LSB Geraldo LHM de Brito Neto J Mathivet T Antila S. Anatomy and function of the vertebral column lymphatic network in mice. Nat Commun. (2019) 10:4594. doi: 10.1038/s41467-019-12568-w 31597914 PMC6785564

[13] Bouta EM Bell RD Rahimi H Xing L Wood RW Bingham CO. Targeting lymphatic function as a novel therapeutic intervention for rheumatoid arthritis. Nat Rev Rheumatol. (2018) 14:94–106. doi: 10.1038/nrrheum.2017.205 29323343 PMC6475908

[14] Acar M Kocherlakota KS Murphy MM Peyer JG Oguro H Inra CN. Deep imaging of bone marrow shows non-dividing stem cells are mainly perisinusoidal. Nature. (2015) 526:126–30. doi: 10.1038/nature15250 PMC4850557 26416744

[15] Ramasamy SK Kusumbe AP Schiller M Zeuschner D Bixel MG Milia C. Blood flow controls bone vascular function and osteogenesis. Nat Commun. (2016) 7:13601. doi: 10.1038/ncomms13601 27922003 PMC5150650

[16] Filipowska J Tomaszewski KA Niediźwiedzki Ł Walocha JA Niedźwiedzki T. The role of vasculature in bone development, regeneration and proper systemic functioning. Angiogenesis. (2017) 20:291–302. doi: 10.1007/s10456-017-9541-1 28194536 PMC5511612

[17] Kusumbe AP Ramasamy SK Itkin T Mäe MA Langen UH Betsholtz C. Age-dependent modulation of vascular niches for haematopoietic stem cells. Nature. (2016) 532:380–4. doi: 10.1038/nature17638 PMC5035541 27074508

[18] Sivaraj KK Adams RH. Blood vessel formation and function in bone. Development. (2016) 143:2706–15. doi: 10.1242/dev.136861 27486231

[19] Arnett TR. Acidosis, hypoxia and bone. Arch Biochem Biophys. (2010) 503:103–9. doi: 10.1016/j.abb.2010.07.021 20655868

[20] Spencer JA Ferraro F Roussakis E Klein A Wu J Runnels JM. Direct measurement of local oxygen concentration in the bone marrow of live animals. Nature. (2014) 508:269–73. doi: 10.1038/nature13034 PMC3984353 24590072

[21] Itkin T Gur-Cohen S Spencer JA Schajnovitz A Ramasamy SK Kusumbe AP. Distinct bone marrow blood vessels differentially regulate haematopoiesis. Nature. (2016) 532:323–8. doi: 10.1038/nature17624 PMC6450701 27074509

[22] Bixel MG Kusumbe AP Ramasamy SK Sivaraj KK Butz S Vestweber D. Flow dynamics and HSPC homing in bone marrow microvessels. Cell Rep. (2017) 18:1804–16. doi: 10.1016/j.celrep.2017.01.042 PMC5318670 28199850

[23] Lo Celso C Lin CP Scadden DT. *In vivo* imaging of transplanted hematopoietic stem and progenitor cells in mouse calvarium bone marrow. Nat Protoc. (2011) 6:1–14. doi: 10.1038/nprot.2010.168 21212779 PMC3382040

[24] Langen UH Pitulescu ME Kim JM Enriquez-Gasca R Sivaraj KK Kusumbe AP. Cell–matrix signals specify bone endothelial cells during developmental osteogenesis. Nat Cell Biol. (2017) 19:189–201. doi: 10.1038/ncb3476 28218908 PMC5580829

[25] Zawieja D. Lymphatic biology and the microcirculation: past, present and future. Microcirculation. (2005) 12:141–50. doi: 10.1080/10739680590900003 15804980

[26] Petrova TV Koh GY. Biological functions of lymphatic vessels. Science. (2020) 369:eaax4063. doi: 10.1126/science.aax4063 32646971

[27] Srinivasan RS Dillard ME Lagutin OV Lin FJ Tsai S Tsai MJ. Lineage tracing demonstrates the venous origin of the mammalian lymphatic vasculature. Genes Dev. (2007) 21:2422–32. doi: 10.1101/gad.1588407 PMC1993873 17908929

[28] Skandalakis JE Skandalakis LJ Skandalakis PN. Anatomy of the lymphatics. Surg Oncol Clin N Am. (2007) 16:1–16. doi: 10.1016/j.soc.2006.10.006 17336233

[29] Yang Y Oliver G. Development of the mammalian lymphatic vasculature. J Clin Invest. (2014) 124:888–97. doi: 10.1172/JCI71609 PMC3938267 24590273

[30] Tammela T Alitalo K. Lymphangiogenesis: molecular mechanisms and future promise. Cell. (2010) 140:460–76. doi: 10.1016/j.cell.2010.01.045 20178740

[31] Breslin JW Yang Y Scallan JP Sweat RS Adderley SP Murfee WL. Lymphatic vessel network structure and physiology. In: Comprehensive Physiology. John Wiley & Sons, Ltd (2018). p. 207–99. doi: 10.1002/cphy.c180015 PMC6459625 30549020

[32] Schulte-Merker S Sabine A Petrova TV. Lymphatic vascular morphogenesis in development, physiology, and disease. J Cell Biol. (2011) 193:607–18. doi: 10.1083/jcb.201012094 PMC3166860 21576390

[33] Zawieja DC. Contractile physiology of lymphatics. Lymphat Res Biol. (2009) 7:87–96. doi: 10.1089/lrb.2009.0007 19534632 PMC2925033

[34] Baluk P Fuxe J Hashizume H Romano T Lashnits E Butz S. Functionally specialized junctions between endothelial cells of lymphatic vessels. J Exp Med. (2007) 204:2349–62. doi: 10.1084/jem.20062596 PMC2118470 17846148

[35] Chakraborty S Davis MJ Muthuchamy M. Emerging trends in the pathophysiology of lymphatic contractile function. Semin Cell Dev Biol. (2015) 38:55–66. doi: 10.1016/j.semcdb.2015.01.005 25617600 PMC4397138

[36] Aspelund A Robciuc MR Karaman S Makinen T Alitalo K. Lymphatic system in cardiovascular medicine. Circ Res. (2016) 118:515–30. doi: 10.1161/CIRCRESAHA.115.306544 26846644

[37] Padera TP Meijer EFJ Munn LL. The lymphatic system in disease processes and cancer progression. Annu Rev BioMed Eng. (2016) 18:125–58. doi: 10.1146/annurev-bioeng-112315-031200 PMC4946986 26863922

[38] Wu Y Seong YJ Li K Choi D Park E Daghlian GH. Organogenesis and distribution of the ocular lymphatic vessels in the anterior eye. JCI Insight. (2020) 5:e135121, 135121. doi: 10.1172/jci.insight.135121 32641580 PMC7406257

[39] Kizhatil K Ryan M Marchant JK Henrich S John SWM. Schlemm’s canal is a unique vessel with a combination of blood vascular and lymphatic phenotypes that forms by a novel developmental process. PloS Biol. (2014) 12:e1001912. doi: 10.1371/journal.pbio.1001912 25051267 PMC4106723

[40] Monroy M McCarter AL Hominick D Cassidy N Dellinger MT. Lymphatics in bone arise from pre-existing lymphatics. Dev Camb Engl. (2020) 147:dev184291. doi: 10.1242/dev.184291 PMC7188445 32188632

[41] Wang W Wang H Zhou X Li X Sun W Dellinger M. Lymphatic endothelial cells produce M-CSF, causing massive bone loss in mice. J Bone Miner Res Off J Am Soc Bone Miner Res. (2017) 32:939–50. doi: 10.1002/jbmr.3077 PMC5413433 28052488

[42] Hominick D Silva A Khurana N Liu Y Dechow PC Feng JQ. VEGF-C promotes the development of lymphatics in bone and bone loss. eLife. (2018) 7:e34323. doi: 10.7554/eLife.34323 29620526 PMC5903859

[43] Edwards JR Williams K Kindblom LG Meis-Kindblom JM Hogendoorn PCW Hughes D. Lymphatics and bone. Hum Pathol. (2008) 39:49–55. doi: 10.1016/j.humpath.2007.04.022 17904616

[44] Dillaman RM. Movement of ferritin in the 2-day-old chick femur. Anat Rec. (1984) 209:445–53. doi: 10.1002/ar.1092090404 6476415

[45] Montgomery RJ Sutker BD Bronk JT Smith SR Kelly PJ. Interstitial fluid flow in cortical bone. Microvasc Res. (1988) 35:295–307. doi: 10.1016/0026-2862(88)90084-2 3393091

[46] Vittas D Hainau B. Lymphatic capillaries of the periosteum: do they exist? Lymphology. (1989) 22:173–7. 2632992

[47] Casley-Smith JR Földi-Börsök E Földi M. The prelymphatic pathways of the brain as revealed by cervical lymphatic obstruction and the passage of particles. Br J Exp Pathol. (1976) 57:179–88. PMC2041107 773400

[48] Louveau A Herz J Alme MN Salvador AF Dong MQ Viar KE. CNS lymphatic drainage and neuroinflammation are regulated by meningeal lymphatic vasculature. Nat Neurosci. (2018) 21:1380–91. https://pubmed.ncbi.nlm.nih.gov/30224810/. 30224810 10.1038/s41593-018-0227-9 PMC6214619

[49] Aspelund A Antila S Proulx ST Karlsen TV Karaman S Detmar M. A dural lymphatic vascular system that drains brain interstitial fluid and macromolecules. J Exp Med. (2015) 212:991–9. doi: 10.1084/jem.20142290 PMC4493418 26077718

[50] Shi JX Liang QQ Wang YJ Mooney RA Boyce BF Xing L. Use of a whole-slide imaging system to assess the presence and alteration of lymphatic vessels in joint sections of arthritic mice. Biotech Histochem Off Publ Biol Stain Commun. (2013) 88:428–39. doi: 10.3109/10520295.2012.729864 PMC3672261 23173750

[51] Melrose J Little CB. Immunolocalization of lymphatic vessels in human fetal knee joint tissues. Connect Tissue Res. (2010) 51:289–305. doi: 10.3109/03008200903318287 20334573

[52] Shi J Liang Q Zuscik M Shen J Chen D Xu H. Distribution and alteration of lymphatic vessels in knee joints of normal and osteoarthritic mice. Arthritis Rheumatol Hoboken NJ. (2014) 66:657–66. doi: 10.1002/art.38278 PMC4074307 24574226

[53] Proulx K Lu A Sumanas S. Cranial vasculature in zebrafish forms by angioblast cluster-derived angiogenesis. Dev Biol. (2010) 348:34–46. doi: 10.1016/j.ydbio.2010.08.036 20832394

[54] Marziano C Genet G Hirschi KK. Vascular endothelial cell specification in health and disease. Angiogenesis. (2021) 24:213–36. doi: 10.1007/s10456-021-09785-7 PMC8205897 33844116

[55] Kim TM Lee RH Kim MS Lewis CA Park C. ETV2/ER71, the key factor leading the paths to vascular regeneration and angiogenic reprogramming. Stem Cell Res Ther. (2023) 14:41. doi: 10.1186/s13287-023-03267-x 36927793 PMC10019431

[56] Lee D Park C Lee H Lugus JJ Kim SH Arentson E. ER71 acts downstream of BMP, Notch, and Wnt signaling in blood and vessel progenitor specification. Cell Stem Cell. (2008) 2:497–507. doi: 10.1016/j.stem.2008.03.008 18462699 PMC2683414

[57] Kelly MA Hirschi KK. Signaling hierarchy regulating human endothelial cell development. Arterioscler Thromb Vasc Biol. (2009) 29:718–24. doi: 10.1161/ATVBAHA.109.184200 PMC2729243 19213939

[58] Sumanas S Lin S. Ets1-related protein is a key regulator of vasculogenesis in zebrafish. PloS Biol. (2006) 4:e10. doi: 10.1371/journal.pbio.0040010 16336046 PMC1310653

[59] Marcelo KL Goldie LC Hirschi KK. Regulation of endothelial cell differentiation and specification. Circ Res. (2013) 112:1272–87. doi: 10.1161/CIRCRESAHA.113.300506 PMC3768127 23620236

[60] Simons M Gordon E Claesson-Welsh L. Mechanisms and regulation of endothelial VEGF receptor signalling. Nat Rev Mol Cell Biol. (2016) 17:611–25. doi: 10.1038/nrm.2016.87 27461391

[61] Wang HU Chen ZF Anderson DJ. Molecular distinction and angiogenic interaction between embryonic arteries and veins revealed by ephrin-B2 and its receptor Eph-B4. Cell. (1998) 93:741–53. doi: 10.1016/S0092-8674(00)81436-1 9630219

[62] Fang JS Coon BG Gillis N Chen Z Qiu J Chittenden TW. Shear-induced Notch-Cx37-p27 axis arrests endothelial cell cycle to enable arterial specification. Nat Commun. (2017) 8:2149. doi: 10.1038/s41467-017-01742-7 29247167 PMC5732288

[63] Masumura T Yamamoto K Shimizu N Obi S Ando J. Shear stress increases expression of the arterial endothelial marker ephrinB2 in murine ES cells via the VEGF-Notch signaling pathways. Arterioscler Thromb Vasc Biol. (2009) 29:2125–31. doi: 10.1161/ATVBAHA.109.193185 19797707

[64] Becker PW Sacilotto N Nornes S Neal A Thomas MO Liu K. An intronic flk1 enhancer directs arterial-specific expression via RBPJ-mediated venous repression. Arterioscler Thromb Vasc Biol. (2016) 36:1209–19. doi: 10.1161/ATVBAHA.116.307517 PMC4894770 27079877

[65] Casie Chetty S Rost MS Enriquez JR Schumacher JA Baltrunaite K Rossi A. Vegf signaling promotes vascular endothelial differentiation by modulating etv2 expression. Dev Biol. (2017) 424:147–61. doi: 10.1016/j.ydbio.2017.03.005 PMC5419721 28279709

[66] Iso T Maeno T Oike Y Yamazaki M Doi H Arai M. Dll4-selective Notch signaling induces ephrinB2 gene expression in endothelial cells. Biochem Biophys Res Commun. (2006) 341:708–14. doi: 10.1016/j.bbrc.2006.01.020 16430858

[67] Hasan SS Fischer A. Notch signaling in the vasculature: angiogenesis and angiocrine functions. Cold Spring Harb Perspect Med. (2023) 13:a041166. doi: 10.1101/cshperspect.a041166 35667708 PMC9899647

[68] Corada M Nyqvist D Orsenigo F Caprini A Giampietro C Taketo MM. The Wnt/beta-catenin pathway modulates vascular remodeling and specification by upregulating Dll4/Notch signaling. Dev Cell. (2010) 18:938–49. doi: 10.1016/j.devcel.2010.05.006 PMC8127076 20627076

[69] García-Pascual CM Zimmermann RC Ferrero H Shawber CJ Kitajewski J Simón C. Delta-like ligand 4 regulates vascular endothelial growth factor receptor 2-driven luteal angiogenesis through induction of a tip/stalk phenotype in proliferating endothelial cells. Fertil Steril. (2013) 100:1768–76. doi: 10.1016/j.fertnstert.2013.08.034 24074756

[70] Jahnsen ED Trindade A Zaun HC Lehoux S Duarte A Jones EAV. Notch1 is pan-endothelial at the onset of flow and regulated by flow. PloS One. (2015) 10:e0122622. doi: 10.1371/journal.pone.0122622 25830332 PMC4382190

[71] You LR Lin FJ Lee CT DeMayo FJ Tsai MJ Tsai SY. Suppression of Notch signalling by the COUP-TFII transcription factor regulates vein identity. Nature. (2005) 435:98–104. doi: 10.1038/nature03511 15875024

[72] Swift MR Weinstein BM. Arterial-venous specification during development. Circ Res. (2009) 104:576–88. doi: 10.1161/CIRCRESAHA.108.188805 19286613

[73] Hong CC Peterson QP Hong JY Peterson RT. Artery/vein specification is governed by opposing phosphatidylinositol-3 kinase and MAP kinase/ERK signaling. Curr Biol CB. (2006) 16:1366–72. doi: 10.1016/j.cub.2006.05.046 PMC1930149 16824925

[74] Lin FJ Chen X Qin J Hong YK Tsai MJ Tsai SY. Direct transcriptional regulation of neuropilin-2 by COUP-TFII modulates multiple steps in murine lymphatic vessel development. J Clin Invest. (2010) 120:1694–707. doi: 10.1172/JCI40101 PMC2860940 20364082

[75] Srinivasan RS Geng X Yang Y Wang Y Mukatira S Studer M. The nuclear hormone receptor Coup-TFII is required for the initiation and early maintenance of Prox1 expression in lymphatic endothelial cells. Genes Dev. (2010) 24:696–707. doi: 10.1101/gad.1859310 20360386 PMC2849126

[76] Aranguren XL Beerens M Coppiello G Wiese C Vandersmissen I Lo Nigro A. COUP-TFII orchestrates venous and lymphatic endothelial identity by homo- or hetero-dimerisation with PROX1. J Cell Sci. (2013) 126:1164–75. doi: 10.1242/jcs.116293 23345397

[77] François M Caprini A Hosking B Orsenigo F Wilhelm D Browne C. Sox18 induces development of the lymphatic vasculature in mice. Nature. (2008) 456:643–7. doi: 10.1038/nature07391 18931657

[78] Chiang IKN Graus MS Kirschnick N Davidson T Luu W Harwood R. The blood vasculature instructs lymphatic patterning in a SOX7-dependent manner. EMBO J. (2023) 42:e109032. doi: 10.15252/embj.2021109032 36715213 PMC9975944

[79] Dieterich LC Klein S Mathelier A Sliwa-Primorac A Ma Q Hong YK. DeepCAGE transcriptomics reveal an important role of the transcription factor MAFB in the lymphatic endothelium. Cell Rep. (2015) 13:1493–504. doi: 10.1016/j.celrep.2015.10.002 26549461

[80] Moreira HR Marques AP. Vascularization in skin wound healing: where do we stand and where do we go? Curr Opin Biotechnol. (2022) 73:253–62. doi: 10.1016/j.copbio.2021.08.019 34555561

[81] Xu X Wang F Yang Y Zhou X Cheng Y Wei X. LIPUS promotes spinal fusion coupling proliferation of type H microvessels in bone. Sci Rep. (2016) 6:20116. doi: 10.1038/srep20116 26830666 PMC4735589

[82] Yan ZQ Wang XK Zhou Y Wang ZG Wang ZX Jin L. H-type blood vessels participate in alveolar bone remodeling during murine tooth extraction healing. Oral Dis. (2020) 26:998–1009. doi: 10.1111/odi.13321 32144839

[83] Chen W Jin X Wang T Bai R Shi J Jiang Y. Ginsenoside Rg1 interferes with the progression of diabetic osteoporosis by promoting type H angiogenesis modulating vasculogenic and osteogenic coupling. Front Pharmacol. (2022) 13:1010937. doi: 10.3389/fphar.2022.1010937 36467080 PMC9712449

[84] Owen-Woods C Kusumbe A. Fundamentals of bone vasculature: Specialization, interactions and functions. Semin Cell Dev Biol. (2022) 123:36–47. doi: 10.1016/j.semcdb.2021.06.025 34281770

[85] Gerber HP Vu TH Ryan AM Kowalski J Werb Z Ferrara N. VEGF couples hypertrophic cartilage remodeling, ossification and angiogenesis during endochondral bone formation. Nat Med. (1999) 5:623–8. doi: 10.1038/9467 10371499

[86] Zhang J Pan J Jing W. Motivating role of type H vessels in bone regeneration. Cell Prolif. (2020) 53:e12874. doi: 10.1111/cpr.12874 33448495 PMC7507571

[87] Rafii S Butler JM Ding BS. Angiocrine functions of organ-specific endothelial cells. Nature. (2016) 529:316–25. doi: 10.1038/nature17040 PMC4878406 26791722

[88] Ramasamy SK Kusumbe AP Adams RH. Regulation of tissue morphogenesis by endothelial cell-derived signals. Trends Cell Biol. (2015) 25:148–57. doi: 10.1016/j.tcb.2014.11.007 PMC4943524 25529933

[89] Romeo SG Alawi KM Rodrigues J Singh A Kusumbe AP Ramasamy SK. Endothelial proteolytic activity and interaction with non-resorbing osteoclasts mediate bone elongation. Nat Cell Biol. (2019) 21:430–41. doi: 10.1038/s41556-019-0304-7 30936475

[90] Maes C Kobayashi T Selig MK Torrekens S Roth SI Mackem S. Osteoblast precursors, but not mature osteoblasts, move into developing and fractured bones along with invading blood vessels. Dev Cell. (2010) 19:329–44. doi: 10.1016/j.devcel.2010.07.010 PMC3540406 20708594

[91] Moya IM Umans L Maas E Pereira PNG Beets K Francis A. Stalk cell phenotype depends on integration of Notch and Smad1/5 signaling cascades. Dev Cell. (2012) 22:501–14. doi: 10.1016/j.devcel.2012.01.007 PMC4544746 22364862

[92] Pitulescu ME Schmidt I Giaimo BD Antoine T Berkenfeld F Ferrante F. Dll4 and Notch signalling couples sprouting angiogenesis and artery formation. Nat Cell Biol. (2017) 19:915–27. doi: 10.1038/ncb3555 28714968

[93] Hasan SS Tsaryk R Lange M Wisniewski L Moore JC Lawson ND. Endothelial Notch signalling limits angiogenesis via control of artery formation. Nat Cell Biol. (2017) 19:928–40. doi: 10.1038/ncb3574 PMC5534340 28714969

[94] Wilgus TA Ferreira AM Oberyszyn TM Bergdall VK Dipietro LA. Regulation of scar formation by vascular endothelial growth factor. Lab Investig J Tech Methods Pathol. (2008) 88:579–90. doi: 10.1038/labinvest.2008.36 PMC2810253 18427552

[95] Han YH Onufer EJ Huang LH Sprung RW Davidson WS Czepielewski RS. Enterically derived high-density lipoprotein restrains liver injury through the portal vein. Science. (2021) 373:eabe6729. doi: 10.1126/science.abe6729 34437091 PMC8478306

[96] Cao E Watt MJ Nowell CJ Quach T Simpson JS De Melo Ferreira V. Mesenteric lymphatic dysfunction promotes insulin resistance and represents a potential treatment target in obesity. Nat Metab. (2021) 3:1175–88. doi: 10.1038/s42255-021-00457-w 34545251

[97] Liu X Cui K Wu H Li KS Peng Q Wang D. Promoting lymphangiogenesis and lymphatic growth and remodeling to treat cardiovascular and metabolic diseases. Arterioscler Thromb Vasc Biol. (2023) 43:e1–10. doi: 10.1161/ATVBAHA.122.318406 36453280 PMC9780193

[98] Zhang F Zarkada G Han J Li J Dubrac A Ola R. Lacteal junction zippering protects against diet-induced obesity. Science. (2018) 361:599–603. doi: 10.1126/science.aap9331 30093598 PMC6317738

[99] Bartok B Firestein GS. Fibroblast-like synoviocytes: key effector cells in rheumatoid arthritis. Immunol Rev. (2010) 233:233–55. doi: 10.1111/j.0105-2896.2009.00859.x PMC2913689 20193003

[100] Smolen JS. Rheumatoid arthritis Primer - behind the scenes. Nat Rev Dis Primer. (2020) 6:32. doi: 10.1038/s41572-020-0168-y 32327647

[101] McInnes IB Schett G. The pathogenesis of rheumatoid arthritis. N Engl J Med. (2011) 365:2205–19. doi: 10.1056/NEJMra1004965 22150039

[102] Carmeliet P Jain RK. Molecular mechanisms and clinical applications of angiogenesis. Nature. (2011) 473:298–307. https://pubmed.ncbi.nlm.nih.gov/21593862/. 21593862 10.1038/nature10144 PMC4049445

[103] Shibuya M Claesson-Welsh L. Signal transduction by VEGF receptors in regulation of angiogenesis and lymphangiogenesis. Exp Cell Res. (2006) 312:549–60. https://pubmed.ncbi.nlm.nih.gov/16336962/. 16336962 10.1016/j.yexcr.2005.11.012

[104] Melincovici CS Boşca AB Şuşman S Mărginean M Mihu C Istrate M. Vascular endothelial growth factor (VEGF) - key factor in normal and pathological angiogenesis. Romanian J Morphol Embryol Rev Roum Morphol Embryol. (2018) 59:455–67. https://pubmed.ncbi.nlm.nih.gov/30173249/. 30173249

[105] Bouta EM Kuzin I de Mesy Bentley K Wood RW Rahimi H Ji RC. Brief report: treatment of tumor necrosis factor-transgenic mice with anti-tumor necrosis factor restores lymphatic contractions, repairs lymphatic vessels, and may increase monocyte/macrophage egress. Arthritis Rheumatol Hoboken NJ. (2017) 69:1187–93. doi: 10.1002/art.40047 PMC5449211 28118521

[106] Ponomarev LC Ksiazkiewicz J Staring MW Luttun A Zwijsen A. The BMP pathway in blood vessel and lymphatic vessel biology. Int J Mol Sci. (2021) 22:6364. doi: 10.3390/ijms22126364 34198654 PMC8232321

[107] Renò F Sabbatini M. Breaking a vicious circle: lymphangiogenesis as a new therapeutic target in wound healing. Biomedicines. (2023) 11:656. doi: 10.3390/biomedicines11030656 36979635 PMC10045303

[108] Wang Y Jin Y Mäe MA Zhang Y Ortsäter H Betsholtz C. Smooth muscle cell recruitment to lymphatic vessels requires PDGFB and impacts vessel size but not identity. Dev Camb Engl. (2017) 144:3590–601. doi: 10.1242/dev.147967 PMC5665477 28851707

[109] Onimaru M Yonemitsu Y Fujii T Tanii M Nakano T Nakagawa K. VEGF-C regulates lymphangiogenesis and capillary stability by regulation of PDGF-B. Am J Physiol Heart Circ Physiol. (2009) 297:H1685–1696. doi: 10.1152/ajpheart.00015.2009 19734356

[110] Kurashige C Hosono K Matsuda H Tsujikawa K Okamoto H Majima M. Roles of receptor activity-modifying protein 1 in angiogenesis and lymphangiogenesis during skin wound healing in mice. FASEB J Off Publ Fed Am Soc Exp Biol. (2014) 28:1237–47. doi: 10.1096/fj.13-238998 24308973

[111] Gerald D Adini I Shechter S Perruzzi C Varnau J Hopkins B. RhoB controls coordination of adult angiogenesis and lymphangiogenesis following injury by regulating VEZF1-mediated transcription. Nat Commun. (2013) 4:2824. doi: 10.1038/ncomms3824 24280686 PMC3868161

[112] Naiche LA Villa SR Kitajewski JK. Endothelial cell fate determination: A top notch job in vascular decision-making. Cold Spring Harb Perspect Med. (2022) 12:a041183. doi: 10.1101/cshperspect.a041183 35288401 PMC9619357

[113] Okizaki SI Ito Y Hosono K Oba K Ohkubo H Kojo K. Vascular endothelial growth factor receptor type 1 signaling prevents delayed wound healing in diabetes by attenuating the production of IL-1β by recruited macrophages. Am J Pathol. (2016) 186:1481–98. doi: 10.1016/j.ajpath.2016.02.014 27085138

[114] Lim L Bui H Farrelly O Yang J Li L Enis D. Hemostasis stimulates lymphangiogenesis through release and activation of VEGFC. Blood. (2019) 134:1764–75. doi: 10.1182/blood.2019001736 PMC6856989 31562136

[115] Tabata M Kadomatsu T Fukuhara S Miyata K Ito Y Endo M. Angiopoietin-like protein 2 promotes chronic adipose tissue inflammation and obesity-related systemic insulin resistance. Cell Metab. (2009) 10:178–88. doi: 10.1016/j.cmet.2009.08.003 19723494

[116] Cho CH Sung HK Kim KT Cheon HG Oh GT Hong HJ. COMP-angiopoietin-1 promotes wound healing through enhanced angiogenesis, lymphangiogenesis, and blood flow in a diabetic mouse model. Proc Natl Acad Sci U S A. (2006) 103:4946–51. doi: 10.1073/pnas.0506352103 PMC1458775 16543381

[117] Hiepen C Mendez PL Knaus P. It takes two to tango: endothelial TGFβ/BMP signaling crosstalk with mechanobiology. Cells. (2020) 9:1965. doi: 10.3390/cells9091965 32858894 PMC7564048

[118] Goumans MJ Zwijsen A Ten Dijke P Bailly S. Bone morphogenetic proteins in vascular homeostasis and disease. Cold Spring Harb Perspect Biol. (2018) 10:a031989. doi: 10.1101/cshperspect.a031989 28348038 PMC5793761

[119] Morrell NW Bloch DB ten Dijke P Goumans MJTH Hata A Smith J. Targeting BMP signalling in cardiovascular disease and anaemia. Nat Rev Cardiol. (2016) 13:106–20. doi: 10.1038/nrcardio.2015.156 PMC4886232 26461965

[120] García de Vinuesa A Abdelilah-Seyfried S Knaus P Zwijsen A Bailly S. BMP signaling in vascular biology and dysfunction. Cytokine Growth Factor Rev. (2016) 27:65–79. doi: 10.1016/j.cytogfr.2015.12.005 26823333

[121] Subileau M Merdzhanova G Ciais D Collin-Faure V Feige JJ Bailly S. Bone morphogenetic protein 9 regulates early lymphatic-specified endothelial cell expansion during mouse embryonic stem cell differentiation. Stem Cell Rep. (2019) 12:98–111. doi: 10.1016/j.stemcr.2018.11.024 PMC6335586 30595547

[122] Levet S Ciais D Merdzhanova G Mallet C Zimmers TA Lee SJ. Bone morphogenetic protein 9 (BMP9) controls lymphatic vessel maturation and valve formation. Blood. (2013) 122:598–607. doi: 10.1182/blood-2012-12-472142 23741013 PMC3724195

[123] Chen H Brady Ridgway J Sai T Lai J Warming S Chen H. Context-dependent signaling defines roles of BMP9 and BMP10 in embryonic and postnatal development. Proc Natl Acad Sci U S A. (2013) 110:11887–92. doi: 10.1073/pnas.1306074110 PMC3718114 23812757

[124] Yoshimatsu Y Lee YG Akatsu Y Taguchi L Suzuki HI Cunha SI. Bone morphogenetic protein-9 inhibits lymphatic vessel formation via activin receptor-like kinase 1 during development and cancer progression. Proc Natl Acad Sci U S A. (2013) 110:18940–5. doi: 10.1073/pnas.1310479110 PMC3839734 24133138

[125] Dunworth WP Cardona-Costa J Bozkulak EC Kim JD Meadows S Fischer JC. Bone morphogenetic protein 2 signaling negatively modulates lymphatic development in vertebrate embryos. Circ Res. (2014) 114:56–66. doi: 10.1161/CIRCRESAHA.114.302452 24122719 PMC4047637

[126] de Ciriza CP Lawrie A Varo N. OPG expression on endothelial cells and modulation by IL-1B, PDGF, insulin, and glucose. Biochem Physiol Open Access. (2015) 4:1. doi: 10.4172/2168-9652

[127] Basic-Jukic N Gulin M Hudolin T Kastelan Z Katalinic L Coric M. Expression of BMP-2 in vascular endothelial cells of recipient may predict delayed graft function after renal transplantation. Kidney Blood Press Res. (2016) 41:781–93. doi: 10.1159/000450568 27832657

[128] Poulos MG Ramalingam P Gutkin MC Kleppe M Ginsberg M Crowley MJP. Endothelial-specific inhibition of NF-κB enhances functional haematopoiesis. Nat Commun. (2016) 7:13829. doi: 10.1038/ncomms13829 28000664 PMC5187502

[129] Kenswil KJG Jaramillo AC Ping Z Chen S Hoogenboezem RM Mylona MA. Characterization of endothelial cells associated with hematopoietic niche formation in humans identifies IL-33 as an anabolic factor. Cell Rep. (2018) 22:666–78. doi: 10.1016/j.celrep.2017.12.070 29346765

[130] Dainese P Wyngaert KV De Mits S Wittoek R Van Ginckel A Calders P. Association between knee inflammation and knee pain in patients with knee osteoarthritis: a systematic review. Osteoarthritis Cartilage. (2022) 4):516–34. doi: 10.1016/j.joca.2021.12.003 34968719

[131] Sanchez-Lopez E Coras R Torres A Lane NE Guma M. Synovial inflammation in osteoarthritis progression. Nat Rev Rheumatol. (2022) 18:258–75. doi: 10.1038/s41584-022-00749-9 PMC9050956 35165404

[132] Yang CY Chanalaris A Troeberg L. ADAMTS and ADAM metalloproteinases in osteoarthritis - looking beyond the ‘usual suspects’. Osteoarthritis Cartilage. (2017) 25:1000–9. doi: 10.1016/j.joca.2017.02.791 PMC5473942 28216310

[133] Grillet B Pereira RVS Van Damme J Abu El-Asrar A Proost P Opdenakker G. Matrix metalloproteinases in arthritis: towards precision medicine. Nat Rev Rheumatol. (2023) 19:363–77. doi: 10.1038/s41584-023-00966-w 37161083

[134] Xu H Edwards J Banerji S Prevo R Jackson DG Athanasou NA. Distribution of lymphatic vessels in normal and arthritic human synovial tissues. Ann Rheum Dis. (2003) 62:1227–9. doi: 10.1136/ard.2003.005876 PMC1754407 14644866

[135] Borodin II Liubarskiĭ MS Bgatova NP Mustafaev NR Dremov EI. Morphological criteria of the state of the microcirculation and the lymphatic drainage in the synovial membrane of the knee joint under normal and pathological conditions. Morfol St Petersburg Russ. (2008) 133:51–5. 19069416

[136] Walsh DA Verghese P Cook GJ McWilliams DF Mapp PI Ashraf S. Lymphatic vessels in osteoarthritic human knees. Osteoarthritis Cartilage. (2012) 20:405–12. doi: 10.1016/j.joca.2012.01.012 22326896

[137] Lin X Bell RD Catheline SE Takano T McDavid A Jonason JH. Targeting synovial lymphatic function as a novel therapeutic intervention for age-related osteoarthritis in mice. Arthritis Rheumatol Hoboken NJ. (2023) 75:923–36. doi: 10.1002/art.42441 PMC10238595 36625730

[138] Wu CL Harasymowicz NS Klimak MA Collins KH Guilak F. The role of macrophages in osteoarthritis and cartilage repair. Osteoarthritis Cartilage. (2020) 28:544–54. doi: 10.1016/j.joca.2019.12.007 PMC7214213 31926267

[139] Thomson A Hilkens CMU. Synovial macrophages in osteoarthritis: the key to understanding pathogenesis? Front Immunol. (2021) 12:678757. doi: 10.3389/fimmu.2021.678757 34211470 PMC8239355

[140] Wang W Lin X Xu H Sun W Bouta EM Zuscik MJ. Attenuated joint tissue damage associated with improved synovial lymphatic function following treatment with bortezomib in a mouse model of experimental posttraumatic osteoarthritis. Arthritis Rheumatol Hoboken NJ. (2019) 71:244–57. doi: 10.1002/art.40696 PMC6472916 30144298

[141] Lin X Zhang H Boyce BF Xing L. Ubiquitination of interleukin-1α is associated with increased pro-inflammatory polarization of murine macrophages deficient in the E3 ligase ITCH. J Biol Chem. (2020) 295:11764–75. doi: 10.1074/jbc.RA120.014298 PMC7450106 32587089

[142] Lin X Wang W McDavid A Xu H Boyce BF Xing L. The E3 ubiquitin ligase Itch limits the progression of post-traumatic osteoarthritis in mice by inhibiting macrophage polarization. Osteoarthritis Cartilage. (2021) 29:1225–36. doi: 10.1016/j.joca.2021.04.009 PMC8319075 33940137

[143] Wang W Xu H Sun W Wang H Zuscik M Xing L. Mice deficient in the NF-κB negative regulator, itch, develop severe osteoarthritis and reduced synovial lymphatic drainage due to m1 macrophages-induced lymphatic endothelial cell inflammation. Osteoarthritis Cartilage. (2016) 24:S31–2. doi: 10.1016/j.joca.2016.01.083

[144] Kuang L Wu J Su N Qi H Chen H Zhou S. FGFR3 deficiency enhances CXCL12-dependent chemotaxis of macrophages via upregulating CXCR7 and aggravates joint destruction in mice. Ann Rheum Dis. (2020) 79:112–22. doi: 10.1136/annrheumdis-2019-215696 31662319

[145] Walsh DA McWilliams DF Turley MJ Dixon MR Fransès RE Mapp PI. Angiogenesis and nerve growth factor at the osteochondral junction in rheumatoid arthritis and osteoarthritis. Rheumatol Oxf Engl. (2010) 49:1852–61. doi: 10.1093/rheumatology/keq188 PMC2936950 20581375

[146] Zimmermann-Geller B Köppert S Kesel N Hasseli R Ullrich S Lefèvre S. Interactions between rheumatoid arthritis synovial fibroblast migration and endothelial cells. Immunol Cell Biol. (2019) 97:178–89. doi: 10.1111/imcb.12208 30252968

[147] Wei K Korsunsky I Marshall JL Gao A Watts GFM Major T. Notch signalling drives synovial fibroblast identity and arthritis pathology. Nature. (2020) 582:259–64. doi: 10.1038/s41586-020-2222-z PMC7841716 32499639

[148] Salameh A Galvagni F Bardelli M Bussolino F Oliviero S. Direct recruitment of CRK and GRB2 to VEGFR-3 induces proliferation, migration, and survival of endothelial cells through the activation of ERK, AKT, and JNK pathways. Blood. (2005) 106:3423–31. doi: 10.1182/blood-2005-04-1388 16076871

[149] Xu Y Yuan L Mak J Pardanaud L Caunt M Kasman I. Neuropilin-2 mediates VEGF-C-induced lymphatic sprouting together with VEGFR3. J Cell Biol. (2010) 188:115–30. doi: 10.1083/jcb.200903137 PMC2812843 20065093

[150] Cha HS Bae EK Koh JH Chai JY Jeon CH Ahn KS. Tumor necrosis factor-alpha induces vascular endothelial growth factor-C expression in rheumatoid synoviocytes. J Rheumatol. (2007) 34:16–9. 17216674

[151] Virk MS Lieberman JR. Tumor metastasis to bone. Arthritis Res Ther. (2007) 9 Suppl 1:S5. doi: 10.1186/ar2169 PMC1924520 17634144

[152] Kusumbe AP. Vascular niches for disseminated tumour cells in bone. J Bone Oncol. (2016) 5:112–6. doi: 10.1016/j.jbo.2016.04.003 PMC5063228 27761369

[153] Carlson P Dasgupta A Grzelak CA Kim J Barrett A Coleman IM. Targeting the perivascular niche sensitizes disseminated tumour cells to chemotherapy. Nat Cell Biol. (2019) 21:238–50. doi: 10.1038/s41556-018-0267-0 PMC6948102 30664790

[154] Ghajar CM Peinado H Mori H Matei IR Evason KJ Brazier H. The perivascular niche regulates breast tumour dormancy. Nat Cell Biol. (2013) 15:807–17. doi: 10.1038/ncb2767 PMC3826912 23728425

[155] Sun C Hu A Wang S Tian B Jiang L Liang Y. ADAM17-regulated CX3CL1 expression produced by bone marrow endothelial cells promotes spinal metastasis from hepatocellular carcinoma. Int J Oncol. (2020) 57:249–63. doi: 10.3892/ijo PMC7252465 32319605

[156] Singh A Veeriah V Xi P Labella R Chen J Romeo SG. Angiocrine signals regulate quiescence and therapy resistance in bone metastasis. JCI Insight. (2019) 4:e125679, 125679. doi: 10.1172/jci.insight.125679 31292293 PMC6629249

[157] Heldin CH Vanlandewijck M Moustakas A. Regulation of EMT by TGFβ in cancer. FEBS Lett. (2012) 586:1959–70. doi: 10.1016/j.febslet.2012.02.037 22710176

[158] Brabletz T Kalluri R Nieto MA Weinberg RA. EMT in cancer. Nat Rev Cancer. (2018) 18:128–34. doi: 10.1038/nrc.2017.118 29326430

[159] Padera TP Stoll BR Tooredman JB Capen D di Tomaso E Jain RK. Pathology: cancer cells compress intratumour vessels. Nature. (2004) 427:695. doi: 10.1038/427695a 14973470

[160] Pak KH Park KC Cheong JH. VEGF-C induced by TGF- β1 signaling in gastric cancer enhances tumor-induced lymphangiogenesis. BMC Cancer. (2019) 19:799. doi: 10.1186/s12885-019-5972-y 31409309 PMC6692962

[161] Oka M Iwata C Suzuki HI Kiyono K Morishita Y Watabe T. Inhibition of endogenous TGF-beta signaling enhances lymphangiogenesis. Blood. (2008) 111:4571–9. doi: 10.1182/blood-2007-10-120337 18310502

[162] Baik JE Park HJ Kataru RP Savetsky IL Ly CL Shin J. TGF-β1 mediates pathologic changes of secondary lymphedema by promoting fibrosis and inflammation. Clin Transl Med. (2022) 12:e758. doi: 10.1002/ctm2.758 35652284 PMC9160979

[163] Maertens L Erpicum C Detry B Blacher S Lenoir B Carnet O. Bone marrow-derived mesenchymal stem cells drive lymphangiogenesis. PloS One. (2014) 9:e106976. doi: 10.1371/journal.pone.0106976 25222747 PMC4164522

[164] Conrad C Niess H Huss R Huber S von Luettichau I Nelson PJ. Multipotent mesenchymal stem cells acquire a lymphendothelial phenotype and enhance lymphatic regeneration in *vivo* . Circulation. (2009) 119:281–9. doi: 10.1161/CIRCULATIONAHA.108.793208 19118255

[165] Zhan J Li Y Yu J Zhao Y Cao W Ma J. Culture medium of bone marrow-derived human mesenchymal stem cells effects lymphatic endothelial cells and tumor lymph vessel formation. Oncol Lett. (2015) 9:1221–6. doi: 10.3892/ol.2015.2868 PMC4315037 25663886

[166] Chen RR Silva EA Yuen WW Mooney DJ. Spatio-temporal VEGF and PDGF delivery patterns blood vessel formation and maturation. Pharm Res. (2007) 24:258–64. doi: 10.1007/s11095-006-9173-4 17191092

[167] Kent Leach J Kaigler D Wang Z Krebsbach PH Mooney DJ. Coating of VEGF-releasing scaffolds with bioactive glass for angiogenesis and bone regeneration. Biomaterials. (2006) 27:3249–55. doi: 10.1016/j.biomaterials.2006.01.033 16490250

[168] Quinlan E López-Noriega A Thompson EM Hibbitts A Cryan SA O’Brien FJ. Controlled release of vascular endothelial growth factor from spray-dried alginate microparticles in collagen-hydroxyapatite scaffolds for promoting vascularization and bone repair. J Tissue Eng Regener Med. (2017) 11:1097–109. doi: 10.1002/term.2013 25783558

[169] Kim BS Kim JS Yang SS Kim HW Lim HJ Lee J. Angiogenin-loaded fibrin/bone powder composite scaffold for vascularized bone regeneration. Biomater Res. (2015) 19:18. doi: 10.1186/s40824-015-0040-4 26331087 PMC4552407

[170] Zhou Q Guo R Wood R Boyce BF Liang Q Wang YJ. Vascular endothelial growth factor C attenuates joint damage in chronic inflammatory arthritis by accelerating local lymphatic drainage in mice. Arthritis Rheumatol. (2011) 63:2318–28. doi: 10.1002/art.30421 PMC3149728 21538325

[171] Joukov V Kumar V Sorsa T Arighi E Weich H Saksela O. A recombinant mutant vascular endothelial growth factor-C that has lost vascular endothelial growth factor receptor-2 binding, activation, and vascular permeability activities. J Biol Chem. (1998) 273:6599–602. doi: 10.1074/jbc.273.12.6599 9506953

[172] Yin S Zhang W Zhang Z Jiang X. Recent advances in scaffold design and material for vascularized tissue-engineered bone regeneration. Adv Healthc Mater. (2019) 8:e1801433. doi: 10.1002/adhm.201801433 30938094

[173] Orlandini M Spreafico A Bardelli M Rocchigiani M Salameh A Nucciotti S. Vascular endothelial growth factor-D activates VEGFR-3 expressed in osteoblasts inducing their differentiation. J Biol Chem. (2006) 281:17961–7. doi: 10.1074/jbc.M600413200 16624815

[174] Hartiala P Suominen S Suominen E Kaartinen I Kiiski J Viitanen T. Phase 1 lymfactin® Study: short-term safety of combined adenoviral VEGF-C and lymph node transfer treatment for upper extremity lymphedema. J Plast Reconstr Aesthetic Surg JPRAS. (2020) 73:1612–21. doi: 10.1016/j.bjps.2020.05.009 32513642

